# Effects of Landscape Characteristic Perception of Campus on College Students’ Mental Restoration

**DOI:** 10.3390/bs15040470

**Published:** 2025-04-05

**Authors:** Wei Gao, Binglin Martin Tang, Bing Liu

**Affiliations:** 1 Faculty of Innovation and Design, City University of Macau, Macao 999078, China; u21092120061@cityu.edu.mo; 2 Faculty of International Tourism and Management, City University of Macau, Macao 999078, China; t22092110057@cityu.edu.mo; 3 College of Architecture and Urban Planning, Tongji University, Shanghai 200092, China

**Keywords:** CGS, landscape characteristics, landscape preference, perceived restorativeness, mental restoration

## Abstract

Emerging evidence underscores the beneficial effects of campus green spaces (CGSs) on student well-being and recovery. Previous research has predominantly examined the independent roles of landscape characteristics and preferences in mental restoration. However, limited studies have explored the complex interrelationships among restorative effects, landscape characteristics, preferences, and place-bonding factors, particularly within the context of CGSs. To address this gap, this study developed a validated campus landscape perception scale comprising three dimensions (perception of natural characteristics, perception of artificial characteristics, spatial perception) and 20 related indicators. In the second phase, the scale was used to investigate the influence mechanism of perceived campus landscape characteristics on mental restoration. A total of 36 CGSs across six higher education institutions in Nanjing were selected, representing diverse spatial types. The restoration experiences of 759 participants were measured using psychological indicators when viewing these landscapes. With the help of deep learning techniques, landscape elements were integrated with perceptual factors for partial least squares (PLS)-based statistical analysis. Our findings indicate that the natural and spatial dimensions significantly influence mental restoration, whereas the artificial dimension does not directly impact psychological health. Nevertheless, all dimensions indirectly enhance mental restoration through landscape preference and perceived restorativeness. The study also revealed the moderating effect of objective landscape elements on the relationship between the perception of landscape characteristics and landscape preference. This study confirms the positive role of perceived campus landscape characteristics in fostering mental restoration among students and elucidates the intricate pathway of influence, namely “perception of landscape characteristics → landscape preference → perceived restorativeness → mental restoration”. These findings offer new insights into the complex processes of environmental restoration, where psychological and physical factors are intertwined. Finally, theoretical and managerial implications for improving landscape planning in restoration research are proposed.

## 1. Introduction

During the transition from adolescence to adulthood, university students are exposed to a myriad of pressures, including those related to academic performance, interpersonal relationships, and financial independence. The increased pressure renders them more susceptible to psychological strains like stress, anxiety, mental exhaustion, and depression (Auerbach et al., 2018). According to *The Stressed Years of Their Lives*, the stress associated with college campus life may be at an all-time high (Rostain, 2019). A survey by the WHO has uncovered the prevalence of mental health problems among higher education students, including depression (Akhtar et al., 2020; Huang et al., 2018; Lattie et al., 2019), anxiety (Gong et al., 2023; Harrer et al., 2019; Lattie et al., 2019), stress (Dessauvagie et al., 2022; Gardani et al., 2022; Gong et al., 2023), eating disorders (Harrer et al., 2019, 2020), suicidal ideation (Dessauvagie et al., 2022; Elena et al., 2021), and post-traumatic stress disorder (PTSD) (Barnett et al., 2021; Huang et al., 2018). The underlying factors contributing to these psychological problems may be complex (Elena et al., 2021).

The healing benefits of the environment on individuals were identified as early as 1983. Two predominant theories suggest that exposure to nature plays a restorative role in human health: attention recovery theory (ART) and stress reduction theory (SRT). ART, proposed by Kaplan (R. Kaplan et al., 1989), suggests that the restorativeness of the natural environment is mainly reflected in the restoration of attention. Engaging in non-directed attention activities (observing nature) without the need for mental exertion will have a recovery effect on directed attention fatigue (work and study). SRT, introduced by Ulrich (1984, 1991), suggests that natural environments can alleviate stress and help calm excitement, facilitating a swift recovery from short-term stress. Both theories are grounded in the human’s innate perceptual responses to nature (R. Kaplan, 1989). Based on restorative theory, many scholars have found that interacting with nature can reduce stress, elevate mood, and support mental health (Herzog et al., 1997; Scopelliti & Vittoria Giuliani, 2004; Zhou et al., 2022). Previous studies focused on specific populations, including children and the elderly. However, as mental health problems among college students have come to the forefront, the role of campus surroundings in safeguarding students’ physical and mental well-being has increasingly come under the spotlight (McMahan & Estes, 2015). Based on SRT and ART, numerous studies have explored the relationship between campus green spaces (CGSs) and students’ health, with a particular emphasis on mental health outcomes. Most studies have proved that CGSs have a positive effect on students’ mental health, mainly focusing on four aspects, these being stress relief, attention restoration, emotional improvement, and the enhancement of quality of life and well-being. However, there are differences in the impacts of environmental characteristics of different types and elements (Foellmer et al., 2021; Ha & Kim, 2021; Q. Liu et al., 2022). Despite the growing recognition of the role of CGSs in promoting health and well-being, research on the restorative pathways of CGSs remains underdeveloped.

### 1.1. Landscape Perception and Landscape Preference

**Figure 1:**
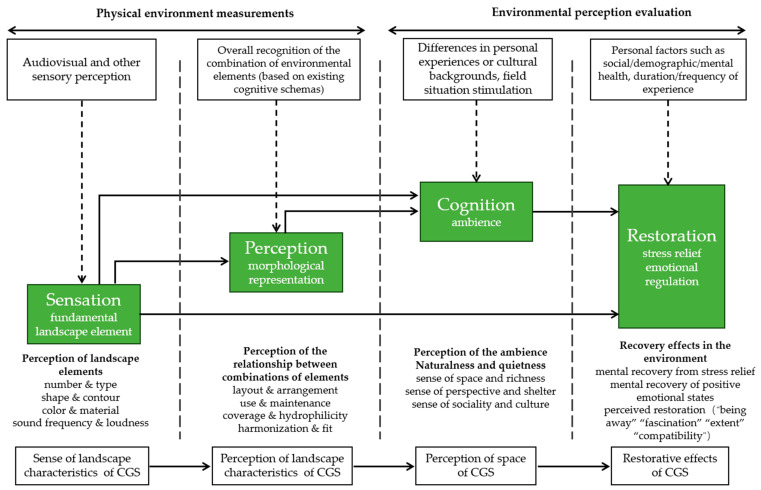
A psychological process-based framework for the study of environmental perception.

Landscape perception refers to a series of processes including a person’s awareness, sensation, attention, and perception of the surrounding environment, and it serves as the foundation for the interaction between humans and the environment (Zurawicki, 2010). The process of the human perception of landscapes is based on multi-sensory experiences, among which visual perception is the main way to obtain information (Ha & Kim, 2021). Perceptual measures are instrumental in reaching consensus on landscape characteristics, as the physical features of an environment do not always align with its restorative potential. These measures provide insight into environmental aspects that cannot be measured directly and physically (Wohlwill, 1976). Environmental psychology divides the process of environmental perception into four steps, i.e., “sensation-perception-cognition-restoration”. (1) Sensation: the process in which human sensory organs receive stimuli from the environment; (2) perception: on the basis of sensation, the sensory stimuli are initially compared with the cognitive schema in the brain, facilitated by knowledge and experience, leading to recognition and formation of a comprehensive image of the object; (3) cognition: the perception and the perceiver’s cultural background, the scene, and the cognitive abilities are integrated, resulting in emotional processing and logical reasoning; (4) restoration: individuals may internalize the perceived environmental information or respond to it through psychological and physiological reactions, influenced by their interests, purposes, needs, values and social norms. Environmental perception is the conversion of the objective physical environment into a subjective experience characterized by atmosphere, emotions, attitudes, and behaviors. This conversion is mediated by the human perception system, which integrates the subject’s memory and imagination. This study will use this framework of environmental perception as a theoretical foundation (Figure 1).

Landscape perception refers to the immediate cognitive evaluation of environmental features (e.g., vegetation density), while landscape preference reflects individuals’ esthetic and functional expectations shaped by long-term experiences (R. Kaplan, 1989). The former is an immediate response to environmental stimuli, while the latter is the expectation of the landscape that develops over time. Landscape preference serves as a widely used perceptual metric, and it is commonly understood as a collective assessment of a locale’s appeal, esthetic caliber, or picturesque allure (Galindo & Hidalgo, 2005). Positive perception depends on whether the information provided by the landscape is easy to understand and encourages the exploration of it. According to ART, being in a pleasant environment helps individuals shift from a state of physiological and psychological tension to a state of relaxation, generating positive impacts and, in turn, bringing about restorative outcomes (R. Kaplan, 1989). Extensive and robust studies have examined the interaction between humans and nature through the lenses of landscape perception and landscape preference. These studies have consistently shown a strong correlation between landscape preference and perceived restorativeness, indicating that environments favored by individuals tend to be more restorative (Eriksson & Nordlund, 2013; Han, 2010; M. Lu & Fu, 2019). Conversely, the inclusion of disliked elements in the environment diminishes its perceived restorativeness. For instance, research indicates that visiting a favored location yields superior restorative outcomes (K. M. Korpela & Ylén, 2009). Additionally, studies reveal that environments with greater preference are more likely to exhibit restorative effects than those that are less favored (Mangone et al., 2021). Therefore, prioritizing and leveraging the preferences of university students as a mediating factor is key to examining the impact of campus environment on their mental restoration.

### 1.2. Role of Perceived Restorativeness in Mental Restoration

The beneficial impact of perceived restorativeness on human well-being has been a topic of long-standing exploration (Nghiem et al., 2021). Based on ART, studies have described perceived restorativeness as an environmental condition for mental restoration experience and better mental health conditions (Martínez-Soto et al., 2014). This concept involves dimensions of “being away” (freeing the mind from everyday life demanding tasks), “fascination” (effortless attention to pleasing objects like birds and flowers), “extent” (occupying the mind for a long period of time), and “compatibility” (a good match with inclinations, no struggle) (R. Kaplan et al., 1989). Perceived restorativeness characteristics can improve mental health by providing relief from stress-related mental disorders (Martínez-Soto et al., 2014). Decades of research have investigated the role of perceived restorativeness in promoting mental restoration, cognitive rehabilitation, and mental fatigue recovery (K. Korpela et al., 2014; Peschardt & Stigsdotter, 2013). Along with an increase in perceived restorativeness, they can lead to an increase in sustained direct attention, stress relief, and recovery of mental fatigue (Felsten, 2009). For instance, in examining the mental health promotion of physical environments, Martínez-Soto et al. (2014) found that individuals exposed to restorative environments exhibit significant improvements in cognitive and emotional functioning, corroborating the mediating role of perceived restorativeness in mental health outcomes. In short, the mechanistic effect of perceived restorativeness is the actual requirement in this process, which can lead to psychological and cognitive restoration (Han, 2003). Several factors have been shown to be related to CGSs’ perceived restorativeness effects, including environmental components and users’ characteristics (Fisher et al., 2021). The current number of empirical studies on campus environments is limited and calls for further investigation. It is imperative to validate the potential mechanism between landscape preference and mental restoration by considering the perceived restorativeness as the mediator. The inclusion of perceived restorativeness enables a more comprehensive understanding of how restoration is facilitated through top-down processes.

Based on the aforementioned theoretical foundations, we developed a model of campus environmental perceived restorativeness linking campus landscape characteristic perception (cognitive evaluation) to landscape preference (affective response), perceived restorativeness (affective response), and ultimately to mental restoration (affective activation outcome).

### 1.3. Moderating Role of Campus Physical Landscape Elements

Green spaces with different landscape features provide important support for individuals’ intrinsic tendencies such as curiosity, interest, seeking challenges, exercising, and developing skills and knowledge in the absence of external rewards (Di Domenico & Ryan, 2017). The physical characteristics of the campus landscape not only affect individuals’ perception of landscape features but also further influence their emotions towards the site (Hartig et al., 1996). Individuals with positive experiences of the site can achieve better psychological outcomes, and this connection is particularly prominent in the context of green spaces, as the physical landscape features of green spaces have potential restorative properties and can satisfy humans’ innate preference for nature (S. Kaplan, 1995). Most current studies use campus physical landscape elements as antecedent variables of mental health. However, after an in-depth analysis of the internal mechanism, this study believes that the moderating effect of landscape elements deserves more attention. For campus green spaces with different spatial characteristics, there will be differences in the relationships among college students’ landscape perception, landscape preference, perceived restorativeness, and mental health.

### 1.4. Aim of the Study

Previous research has largely focused on the relationship between landscape characteristics or preferences and environmental recovery, with studies often examining these two dimensions separately. Consequently, there has been a lack of a cohesive link between landscape characteristics, preferences, and recovery. However, attempting to gauge the preferences–restoration connection without considering the effects of landscape characteristics remains challenging. This oversight leaves the intrinsic landscape characteristics–preference–restoration interplay unexplored. To effectively assess the restorative potential of campus environments, it is essential to integrate the restorative capabilities of landscape elements with landscape preference. This integration is necessary to clarify how landscape characteristics can impact the mental restoration of university students via preferences or other possible perceptual factors.

In conclusion, CGSs hold significant restorative potential, and the mechanism by which CGS landscape characteristics contribute to the restorative benefits for college students has been widely discussed (Guo et al., 2023). However, several gaps remain, namely (1) existing research often focuses on the independent impact of campus landscape characteristics on student mental health, neglecting the interactive effects of other mediating elements and (2) while the significance of landscape preference and perceived restorativeness in explaining the impact of green spaces is well-recognized, limited research has explored the direct and mediating roles of this crucial evaluative metric in the process of students’ mental restoration.

**Figure 2:**
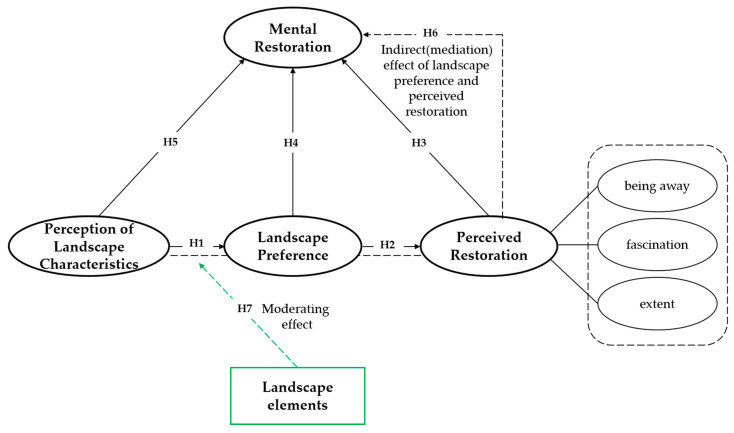
Research model.

To bridge the above gaps, this study examines 36 green spaces across six university campuses in Nanjing by developing a chained mediation model that outlines the impact of CGS environmental perception on students’ mental restoration. As an explorative investigation, the proposed model was tested using the partial least squares method (PLS), a refined structural equation modeling technique known for its comprehensive estimation abilities, surpassing normal multivariate regression models (Ali et al., 2018). Based on the aforementioned theoretical foundations, we proposed hypotheses for direct effects and mediating pathways. Then, the moderating effects of landscape elements were added for testing. The full model was depicted in Figure 2.

**H1.**  *The perception of landscape characteristics has a direct positive effect on landscape preference.*

**H2.**  *Landscape preference has a direct positive effect on perceived restorativeness.*

**H3.**  *Perceived restorativeness has a direct positive effect on mental restoration.*

**H4.**  *Landscape preference has a direct positive effect on mental restoration.*

**H5.**  *The perception of landscape characteristics has a direct positive effect on mental restoration.*

**H6.**  *The effect of the perception of landscape characteristics on mental restoration is chain-mediated by landscape preference and perceived restorativeness.*

**H7.**  *The landscape elements moderate the relationship between the perception of landscape characteristics and landscape preference.*

## 2. Materials and Methods

### 2.1. Research Design

This study included two parts. The first of these was the study focused on the development and testing of the campus landscape perception scale. Due to the lack of an evaluation index system for the perception of campus landscape characteristics, this paper will first propose evaluation indicators for campus landscape characteristics based on existing research. The specific method is to first automatically extract the proportions of landscape elements from a large number of on-site captured images by adopting deep learning technology. These data can reflect the subtle differences in the physical environment of the campus and can assist us in selecting campus spaces with different objective physical landscape features as appropriate research sample points. Then, participants were allowed to view the landscape of the site and answer questions based on their immediate feelings. Moreover, many environmental perception studies simulate outdoor settings and overlay multisensory stimuli to assess subjects’ mental restoration (Browning et al., 2020). While this approach tightly controls external environmental factors affecting subjects’ mental restoration, the process of perceiving environmental features, gaining restorative benefits, and achieving mental restoration typically unfolds in the heterogeneous and complex natural settings of outdoor environments (L. Meng et al., 2022). Therefore, outdoor field experiments are essential for the authenticity and scientific validity of the research.

The collected data were subjected to exploratory factor analysis. Based on the first study and the campus landscape evaluation scale test, a follow-up section was designed to explore the relationships among CGS landscape characteristics perception, landscape preference, perceived restorativeness, and mental restoration. Both surveys were conducted in six colleges and universities in Nanjing, and these campuses were selected because they were built in different periods with varying degrees of naturalness, as will be explained later. The data on the proportions of landscape elements such as water, trees, and the sky at each sampling point obtained through deep learning technology may be the key variables for the moderating effect test in this study.

### 2.2. Study Sites and Materials

**Figure 3:**
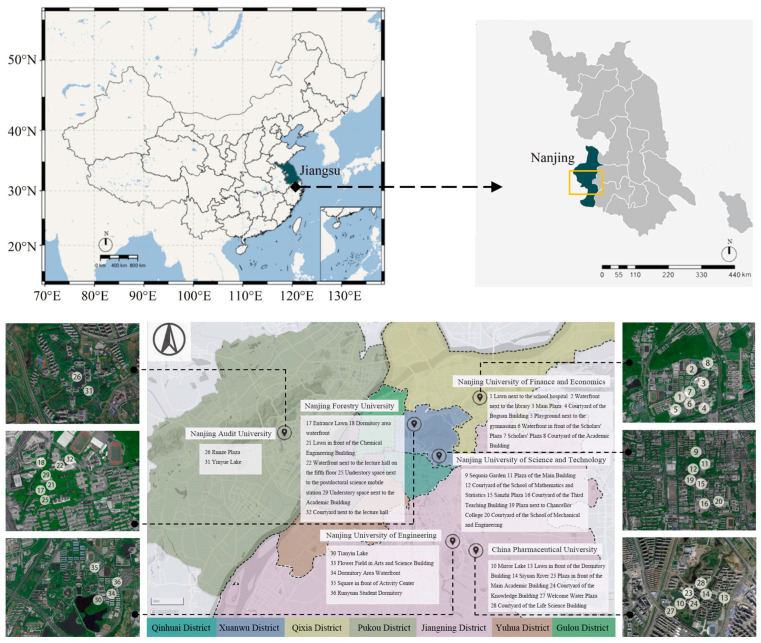
Research model schematic diagram of research area and sample site location. A total of 36 study plots were selected (represented by white points).

**Figure 4:**
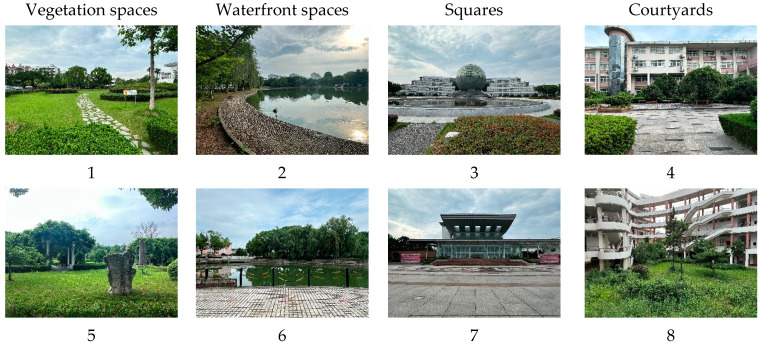

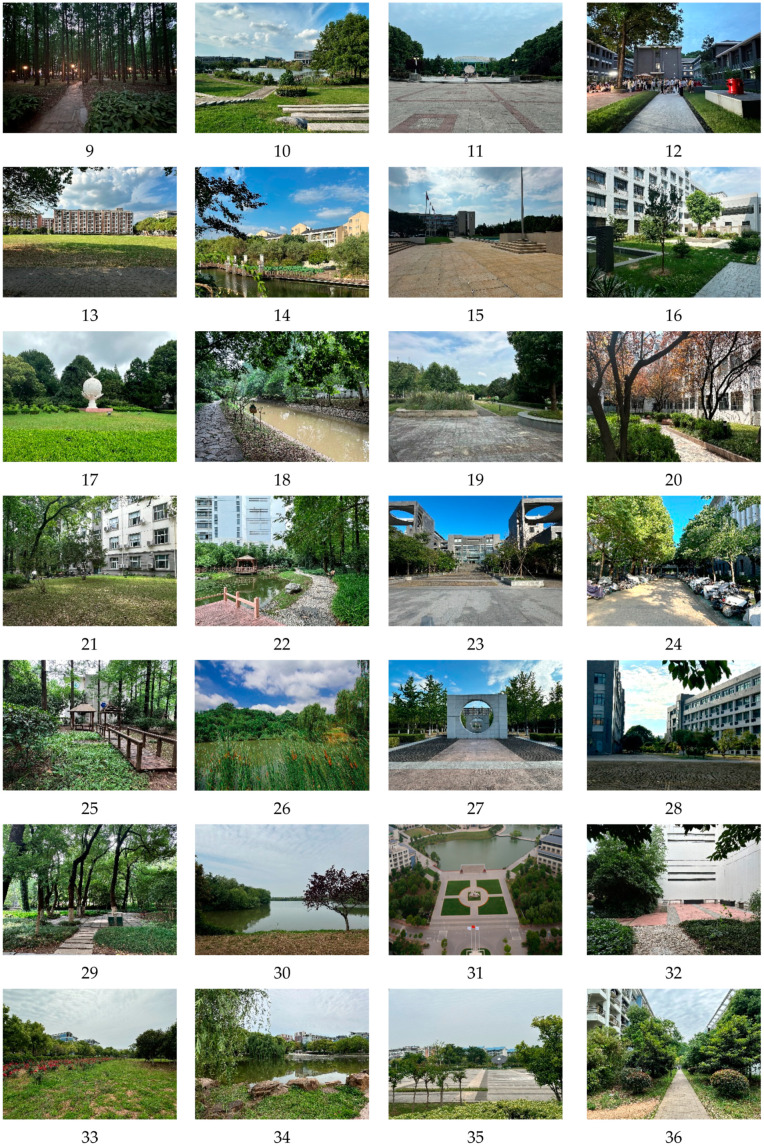
Images of the 36 landscape sites. Pictures were taken by the first author.

Nanjing is home to numerous higher education institutions, with campuses adhering to uniform educational policies and sharing similar climatic conditions; moreover, the student bodies exhibit analogous behavioral patterns. Hence, Nanjing’s university campuses serve as a quintessential subject for evaluation. We first conducted an on-site investigation of 15 universities in Nanjing city. The following criteria had to be met when selecting universities: (1) these universities boast a diverse and representative student body, encompassing a range of characteristics including both old and new campuses; (2) the universities are located in different areas of Nanjing to ensure the representativeness and universality of the sample; (3) the landscape characteristics are obvious and can better reflect the characteristics of various campus spaces; (4) the campuses’ internal landscape elements are diverse, with ample open areas that capture a broad spectrum of visual factors, suitable for the assessment of visual elements; and (5) the flow of students is large and the frequency of use is high. Finally, a total of six institutions were selected for study, including the Nanjing University of Science and Technology, the Nanjing University of Finance and Economics (Qixia Campus), China Pharmaceutical University (Jiangning Campus), Nanjing Forestry University, Nanjing Audit University, and the Nanjing University of Engineering (Table 1). These surveys covered four urban districts, Xuanwu, Jiangning, Qixia, and Pukou. In the present study, we chose 36 sample sites, including waterfront spaces, vegetation spaces, squares spaces, and courtyard spaces; the sites selected for each typology vary somewhat in their composition of landscape elements (Figure 3 and Figure 4).

**Figure 5:**
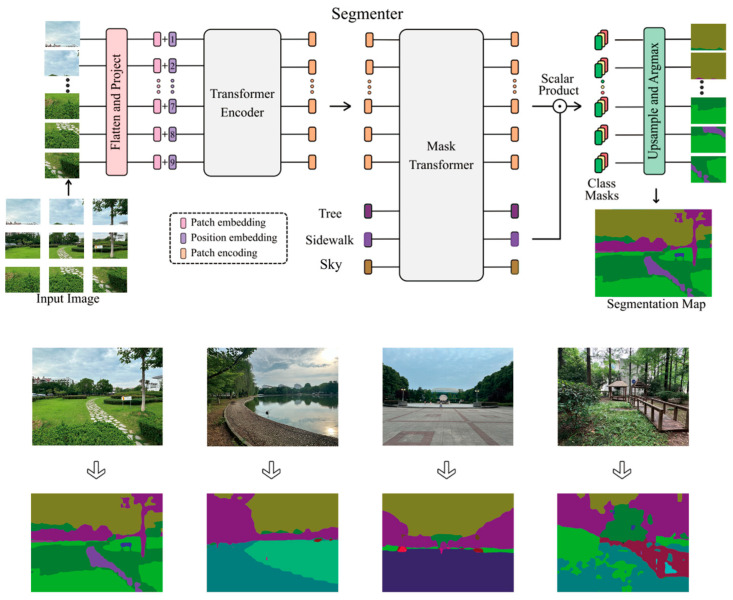
The network structure of deep learning for image segmentation.

When selecting the sample plots among these six campuses, in order to filter the sample plots with distinctive landscape features, we employed a deep learning technique to segment the sample images into common ground objects. Currently, methods based on fully convolutional networks (FCNs) combined with encoder–decoder architectures have become the dominant approach for semantic segmentation (Strudel et al., 2021). Segmentation of images is often ambiguous at the patch level, necessitating contextual cues for consensus on labels. In this study, we employ Segmenter, a transformer-based model for semantic segmentation tasks. Unlike convolution-based methods, this approach models the global context from the outset, leveraging the entire network’s architecture. This approach not only builds on the recent vision transformer (ViT) but also adapts it for semantic segmentation. To achieve this, we utilize the output embeddings of image patches, which we then decode into class labels using either a point-wise linear decoder or a mask transformer decoder. We utilized pre-trained image classification models, fine-tuned them on moderately sized semantic segmentation datasets, and found that this method surpassed FCN-based ones. As Figure 5 shows, major landscape elements were automatically extracted for analysis. The Segmenter model attained over 80% accuracy, satisfying our study’s needs. Finally, we chose seven common landscape characteristics—architecture, sky, trees, lawns, mulching, water, and roads—for statistical analysis to determine the proportion of each in individuals’ visual fields (Table A1).

### 2.3. Part 1 of the Study

#### 2.3.1. Questionnaire Design

The evaluation item of campus environmental perception was selected not only based on previous studies (Q. Liu et al., 2018; J. Ye et al., 2023; K. Li, 2024; Yi & Yang, 2022) but also on the views of professionals in landscape architecture. We engaged five professional architects and landscape architects, along with ten urban planning and landscape architecture students, to suggest 23 evaluative indicators as part of the initial scale to evaluate the environmental feature perception of CGSs. The indicators include fundamental CGS components, including vegetation, animals, water, amenities, and pavements. Indicators pertaining to the overall spatial ambiance were derived from the eight perceived qualities of green spaces (PSDs) put forth by a Swedish University of Agriculture research team in 2010, encompassing “nature”, “culture”, “social”, “prospect”, “rich in species”, “refuge”, “space”, and “serene” (Y. Lu & Liu, 2023). However, “culture” and “space” were excluded from scale development due to students’ semantic comprehension challenges, and “rich in species” was omitted as it was deemed inapplicable to the actual research sites. Respondents were asked to rate their agreement level for each item, from “fully strongly disagree (=1)” to “fully strongly agree (=7)”.

#### 2.3.2. Data Collection

**Figure 6:**
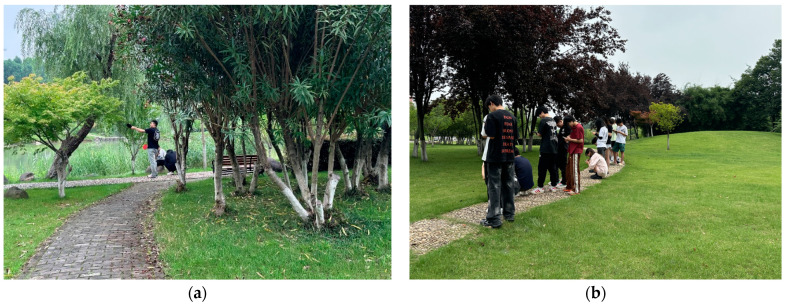
(**a**) Field research 1; (**b**) field research 2.

The pre-survey was undertaken in the green spaces of six universities between approx. 10:00 a.m. and 7:00 p.m. during April 2024 by the research team. The target population comprised the students in the green areas on campus. Participants were guided by the researcher to roam freely within the study sample site and feel the surroundings for 5 min before completing the online questionnaire via an electronic device (Figure 6a,b). We created the online questionnaire using the Star Questionnaires program (https://www.wjx.cn/, date of creation: 9 April 2024), and participants were then asked to scan the QR code generated by the questionnaire system on the spot to fill in the form. Participants were asked to view the scenery of the area and answer the questions based on their immediate feelings. When the questionnaire exercise was finished on site, a small gift valued at USD 2.50 was presented to the participants as a show of appreciation. To ensure that the campus landscape perception scale could effectively reflect students’ perception of the campus landscape, we first distributed 420 questionnaires in the pre-survey and obtained 350 valid samples, with a response rate of 83.3%. Exploratory factor analysis (EFA) was conducted with 350 samples to reduce the number of variables and explore the underlying structure of the principal components.

### 2.4. Part 2 of the Study

#### 2.4.1. Questionnaire Design

The total questionnaire consisted of several parts to measure campus landscape characteristic perception, landscape preference, perceived restorativeness, and mental restoration. Except for the campus landscape characteristic evaluation scale, which was developed by the author, all other scales were adapted from previous studies (Table 2). Measuring items were translated in Chinese to fit the context of campus environments.

For landscape preference, five questions were adapted from previous studies to measure the perceived qualities of the landscape, e.g., “This place has high quality of naturalness”, “The landscape is of liveliness and good visual order”, etc. (Mangone et al., 2021; Ode et al., 2009). Item consistency was tested (Cronbach’s α = 0.921).

For perceived restorativeness, the Perceived Restorativeness Scale (PRS) was used based on a seven-point Likert scale. This scale was developed by Hartig et al. based on the ART and evaluates the perceived restorative quality of an environment in terms of being away, fascination, extent, and compatibility. As there is evidence that condensed dimensions could be available (Hartig et al., 1997), this study only adopted the first three dimensions of the PRS (being away, fascination, and extent) for assessment. Compatibility is the environmental attribute that aligns with an individual’s intentions or favored activities. Given that this study examines students’ environmental perceptions, which are predominantly centered on esthetic rather than active settings, the relevance of the “compatibility” characteristic is comparatively minimal. The PRS does not assess perceived restorative effects. Instead, it measures the restorative qualities perceived in the landscape or environment (Malekinezhad et al., 2020). The results showed high internal consistencies for restorative dimensions (Cronbach’s α = 0.813–0.923).

The mental restoration scale was similarly adapted from previous research to accommodate the realities of university students on CGSs. The quantification of mental health includes the level and the improvement of mental health. The level of mental health has been widely measured using several classical scales, including the general health questionnaire (GHQ-12) (Goldberg & Hillier, 1979), the short Warwick Edinburgh mental well-being scale (Tennant et al., 2007), and the EQ-5D questionnaire (The EuroQol Group, 1990). As our study was designed to investigate immediate psychological improvement following a visit to a campus environment, these instruments were not appropriate. Based on the discovery of two similar studies, we referred to the scale used by Wolf and Wohlfart (2014) and Zhou et al. (2024) to measure immediate mental health benefits, which is generally used for the mental restoration of respondents after receiving environmental stimuli, including dimensions such as improvement in cognition, recovery of mood, and an increase in confidence, which was more suitable for our study. In order to measure immediate mental health benefits after CGS visits, we chose five items. The results showed a high degree of internal consistency between the measures (Cronbach’s α = 0.917).

#### 2.4.2. Data Collection

Participants, all university students from Nanjing, were evenly recruited, ensuring 30 participants per study sample site. Participating students possessed good eyesight and hearing and were free from conditions like color blindness or hearing loss. All participants had the full right to information and were assured anonymity; they voluntarily committed to participate in the study. The study was conducted between 12 June and 18 June 2024, with data collection taking place from 10:00 a.m. to 1:00 p.m. and from 4:00 p.m. to 7:00 p.m. on sunny, windless days. The 30 participants were organized into four groups, with each group led by a researcher, and they traversed different routes to each sample site to mitigate the impact of participant congestion. Participants, guided by researchers to the sample plots, walked and spent time alone in the area, focusing on observing environmental features and ambient sounds for a 5 min duration before completing and submitting an online questionnaire using a mobile device. A total of 1080 questionnaires were amassed. We set up one filter question to identify invalid samples due to the “brain-dead” choice. If a participant chose the wrong answer, the sample would be considered invalid. Moreover, participants who finished the survey in less than 60 s would also be excluded, since our preliminary examinations showed that finishing all questions carefully needs at least one minute. After removing the invalid questionnaires, 759 valid questionnaires were finally obtained in the formal research, and then the relationships among the four variables of landscape perception, landscape preference, perceived restorativeness, and mental restoration were analyzed by structural equation modeling using the valid data.

## 3. Results

### 3.1. Part 1 of the Study: Scale Development

#### 3.1.1. Exploratory Factor Analysis and Examination

The Bartlett test of sphericity (chi-square = 3967.335, df = 253, Sig = 0.000) and Kaiser–Meyer–Olkin test (KMO = 0.758, greater than 0.7) of the campus landscape evaluation scale showed that the collected data are generally reliable. The EFA was performed by using the principal component method with varimax rotation according to latent root criterion of 1.0 on this scale. Items with loadings lower than 0.5 and with loadings higher than 0.4 on more than one factor were eliminated. “Do you think the plant colors in this site are rich?” (factor loading = 0.470) and “Do you think the spatial functions in this site are rich?” (factor loading = 0.465) were removed from the scale. In component 7, the only item with a factor loading exceeding 0.5 was “Do you think the grounds have great views?”, which led to the consideration of its removal. All of the mentioned procedures were performed using SPSS27.0. Subsequently, a six-factor solution with 20 variables was retained, explaining close to 73.253% of the total variance of the scale. Finally, the Kaiser standardized varimax rotation method was applied to rotate the factor loadings, and the results are presented in Table 3. Each item’s loading on the corresponding factor exceeded 0.7, providing a rationale to conclude that the scale used in this study possesses good structural validity. Based on the characteristics of the indicators for each factor, they were named as follows: perception of vegetation, perception of water, perception of animal diversity, perception of rest, perception of site, and spatial perception. Perceptions of vegetation, water, and animal diversity are fundamental natural elements of the natural world, and these three dimensions can be categorized as perceptions of natural landscape characteristics; perceptions of rest and site are typically recognitions of artificial elements and can be categorized as perceptions of artificial landscape characteristics.

#### 3.1.2. Reliability Test of Questionnaire

Based on the reliability testing, the Cronbach’s α values for the six variables of the campus landscape evaluation scale are 0.906, 0.740, 0.712, 0.800, 0.813, and 0.826, respectively. They all fell above the recommended threshold of 0.7—the recommended value as set by Nunnally—and this indicated that there is a high level of consistency within the variables for the factors. Additionally, the CITC values for the questionnaire are all above 0.5, suggesting a satisfactory correlation among the analyzed items. Furthermore, with the exception of item AC2-2, the Cronbach’s α values for all items after their deletion are lower than before; hence, the retention of all items is justified. Item AC2-2 maintains a Cronbach’s α value above 0.8 both before and after deletion, and therefore can be retained. In summary, the questionnaire demonstrates adequate reliability, making it suitable for further analysis (Table 4).

#### 3.1.3. Correlation Between Landscape Elements and Perception Indicators

**Figure 7:**
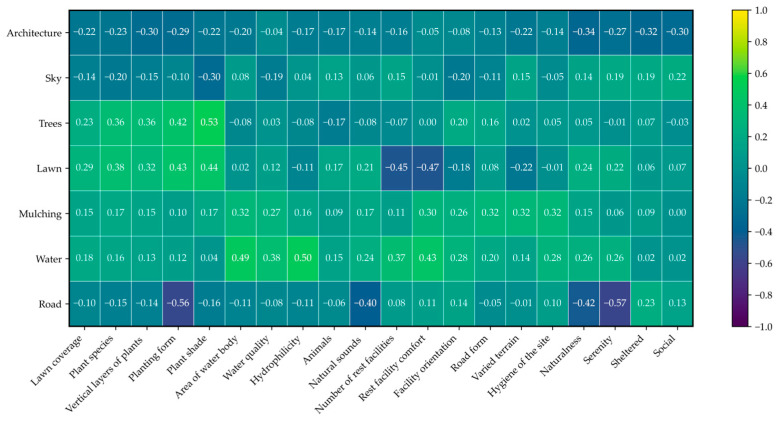
The Pearson correlation coefficients between landscape elements and subjective perception indicators.

In this study, Pearson correlation analysis was used to quantify potential links between subjective perception indicators and objective landscape elements. The results (Figure 7) showed that trees, lawns, water, and roads are key factors influencing college students’ landscape perception. Specifically, trees showed significant positive correlations with students’ subjective perception of natural landscape features, such as plant species (r = 0.36), vertical layers of plants (r = 0.36), planting form (r = 0.42), and plant shade (r = 0.53). This suggests that the richness and diversity of trees can strengthen students’ perception of campus green spaces’ natural features. Water exhibited significant positive correlations with area of water body (r = 0.49), hydrophilicity (r = 0.50), and rest facility comfort (r = 0.43), indicating that water bodies and their hydrophilic designs can boost students’ perception of natural features and resting comfort. Lawns were positively correlated with students’ subjective perception of plants but negatively correlated with the number (r = −0.45) and comfort (r = −0.47) of resting facilities, suggesting that in open plant spaces, these facilities are insufficient to meet student needs. From the analysis, it is clear that natural elements like rich trees, open lawns, and flowing water significantly enhance students’ subjective experience. Roads are negatively correlated with overall perception, especially with planting form (r = −0.56), natural sounds (r = −0.40), naturalness (r = −0.42), and serenity (r = −0.57). This implies roads may weaken students’ positive visual perception of campus green spaces. Similarly, architecture shows a trend of reducing landscape feature perception, stressing the importance of balancing artificial and natural elements in campus green space planning to improve student experience quality.

Based on the data analysis of landscape elements in four types of spaces (waterfront spaces, vegetation spaces, squares, and courtyards), Pearson correlation coefficients were calculated for 20 perception indicators.

(1) Waterfront Spaces

**Figure 8:**
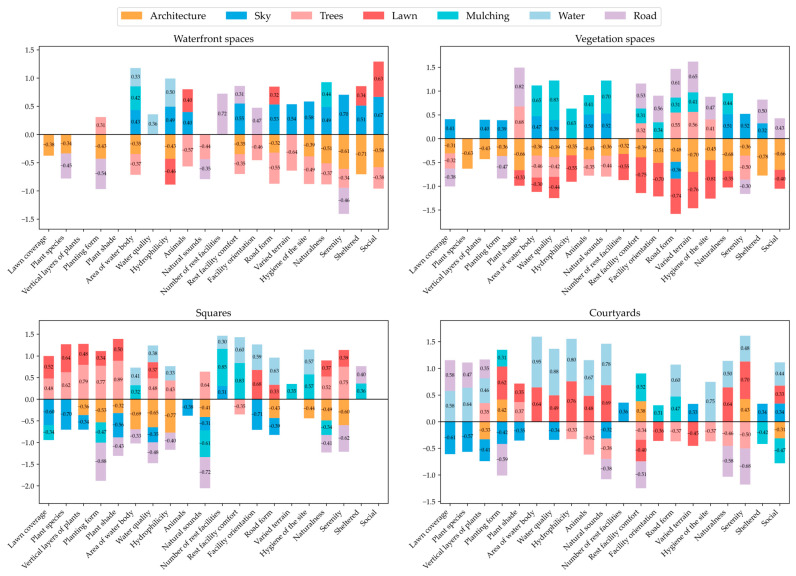
Based on the data analysis of landscape elements in four types of spaces, Pearson correlation coefficients were calculated for the perception indicators.

As shown in Figure 8, for the perception evaluation of waterfront spaces, water, as the main landscape element in these spaces, is significantly positively correlated with indicators such as area of water body and hydrophilicity. This indicates that in waterfront spaces, the presence of water bodies and their hydrophilic designs can significantly enhance the perception of these indicators. Architectures are significantly negatively correlated with indicators such as naturalness and serenity, suggesting that an excessive number of building elements may reduce the naturalness and tranquility of waterfront spaces. Lawns are significantly positively correlated with the social indicator, indicating that lawns can facilitate communication and interaction among college students in waterfront spaces.

(2) Vegetation Spaces

The data in Figure 8 highlight a significant contrast in the perception values between architecture and vegetation in vegetation spaces. In plant-dominated spaces, the richness and diversity of trees and ground covers can significantly enhance the perception of the natural features and quality of green spaces. However, architecture limits visual diversity, leading to monotony and discomfort in individual perception.

(3) Squares

In square spaces, the scores in the natural perception evaluation are generally lower than average, with obvious artificial modification features. Elements such as trees, mulching, and water are significantly positively correlated with college students’ subjective perception of the landscape, emphasizing the positive role of natural landscape elements in attracting people and promoting subjective perception in square spaces. Nevertheless, architecture and roads are negatively correlated with subjective perception, indicating the negative impact of artificial elements on subjective perception in square spaces.

(4) Courtyards

The uniqueness of courtyard spaces lies in their integration of elements such as plants, water, and buildings, creating a rich, beautiful, and pleasant visual experience, which in turn receives high-level evaluations in subjective perception. Figure 8 shows that water plays a significant role in overall perception, especially in dimensions related to natural perception, receiving positive evaluations. Compared with waterfront spaces, college students have a stronger perception of water body elements in courtyard spaces and give higher evaluations of them. Secondly, lawns also play an important role. They are significantly positively correlated with indicators such as natural sounds, naturalness, and *serenity*, indicating that lawns can significantly enhance the perception experience of college students in courtyard spaces.

Through comparative analysis, the differences in subjective perception among different campus space types were identified, and the relationships between key elements and perception indicators were explored in depth, reflecting the effectiveness and areas for improvement in campus landscape design.

### 3.2. Part 2 of the Study: Mental Restorative Effects of Campus

#### 3.2.1. Summary Statistics

A total of 1080 respondents completed the questionnaire in this study, with 759 valid questionnaires (vegetation spaces = 189, waterfront spaces = 178, squares = 202, and courtyards = 190), of which 50.9% were male (n = 386) and 49.1% were female (n = 373); the academic composition covered undergraduate students accounting for 52.8% (n = 401), master’s degree students 37.7% (n = 286), and PhD students 9.5% (n = 72). The questionnaire survey covered a number of faculties such as architecture and urban planning, art and design, civil engineering, business administration, auditing, medicine, pharmacy, etc., where majors related to architecture, planning, and design accounted for 43.1 (n = 327) of all respondents, and other majors accounted for 56.9 (n = 432) of the students. The number of hours spent on studying in the recent week was as follows: 38.9% (n = 295) of the students spent an average of 5–6 h per day, and while most of the participants were able to ensure sufficient sleep (45.3%, n = 344, for an average of 8–9 h per day; 43.9%, n = 333, for an average of 6–7 h per day), some of the students reported that study pressure and emotions such as stress and anxiety led to poorer quality of sleep in the recent week. In the self-stress evaluation, participants perceived that they had occasional bad moods in 37% of cases (n = 281), perceived that they often felt negative and depressed in 21.3% of cases (n = 162), and perceived that stress had seriously affected their lives in 5.3% of cases (n = 40). The participants usually had occasional lack of energy (39.9%, n = 303) or frequent lack of energy problems (32%, n = 243), and the majority of participants wanted to make changes when problems such as stress and inattention arose but did not know by what means (55.2%, n = 419) (Table 5).

Affected by the exam week, compared to normal times, university students in Nanjing had less exercise, more screen browsing, worse sleep, and insufficient social interaction; the detection rates of sleep problems and related physical and mental disorders were also much higher. In existing studies to measure the level of recovery of attention and stress, stimuli sources are usually preset to put respondents in a state of anxiety, stress, and mental fatigue (Ratcliffe & Korpela, 2018). Conducting the test during the exam week eliminates the step of setting up the stimulation sources and avoids the uncertainties brought about by this method.

#### 3.2.2. Descriptive Statistics of Variables

The descriptive statistics of the data (n = 759) for the latent variables are summarized in Table 6. In terms of the perception of landscape characteristics, the mean score of vegetation was 3.43 [Std. ± 1.77], water was 3.90 [Std. ± 1.50], animal diversity was 3.71 [Std. ± 1.57], rest was 3.64 [Std. ± 1.54], site was 3.70 [Std. ± 1.48], and spatial perception was 4.78 [Std. ± 1.30]. The results showed that the mean scores of the perceived measures were as follows: landscape preference was 4.73 [Std. ± 1.28] and mental restoration was 4.66 [Std. ± 1.32]. The standard deviation of each latent variable ranged from 1.26 to 1.77, indicating that the scores of the variables were relatively stable and did not fluctuate much. The mean scores for each variable were above three, indicating that the respondents’ overall evaluation of the six campuses was positive. The descriptive statistics of the observed variables are also presented in Table 6, and the standard deviations for these observed variables range from 8.12 to 12.74.

#### 3.2.3. Common Method Bias Test

Given this study’s reliance on a questionnaire approach, the data for multiple variables are sourced from the same respondents, measurement settings, and questionnaire features, which can be susceptible to common method bias (CMB). Following Podsakoff P M. et al.’s recommendations, the questionnaire was pre-tested twice to ensure quality, with semantic adjustments made based on respondent feedback (Podsakoff et al., 2003). Additionally, questions pertaining to different variables were set across various pages to reduce the probability of respondents forming associations between earlier and later variables. Second, the survey ensured respondent anonymity to mitigate potential biases (M. Meng & Zhu, 2018). Lastly, the collected latent variable data underwent Harman’s one-factor test, yielding 11 factors with eigenvalues exceeding one, and the most substantial factor accounted for 26.691% of the variance (<30%), indicating no significant CMB in this study’s data.

#### 3.2.4. Measurement Model

The measurement model was examined to ensure the construct validity for landscape preference (LP), perception of natural landscape characteristics (NC), perception of artificial landscape characteristics (AC), spatial perception (SA), perceived restorativeness (PRS), and mental restoration (MR). In this regard, several criteria should be met, including the internal consistency of the measurement model (reliability and factor loadings), convergent validity, and discriminant validity.

In terms of the internal consistency of the measurement model, the results indicated that Cronbach ’s α (0.722–0.923) values of the sub-constructs were above the recommended criterion of 0.70, indicating high reliability of the scales corresponding to each variable. For reliability of the measurement items, the factor loadings for all items were above 0.7, indicating that each measurement item has a good explanatory power for the corresponding variable. For second-order constructs, Cronbach’s α (0.810–0.872) and the factor loadings for all measurement items were above 0.6, meeting the recommended standards and indicating acceptable scale consistency. For convergent validity, according to the criteria by Fornell and Larcker (Bagozzi, 1981), when composite reliability (CR) exceeds 0.6 and average variance extracted (AVE) exceeds 0.5, convergent validity is considered optimal. The results indicated that the composite reliability (CR) of all items or sub-constructs was above 0.7 and highly significant at the *p* = 0.001 level, and the average variance extracted (AVE) ranged from 0.525 to 0.812, which was highly above the recommended criterion of 0.50. A summary of the construct validity is presented in Table 7.

Assessing discriminant validity is essential for confirming the distinctness of individual constructs. This study utilized two approaches to assess discriminant validity. The primary method employed was the Fornell–Larcker criterion. Per the Fornell–Larcker criterion, the square root of the AVE for each construct must exceed its correlations with other constructs. Table 8 illustrates that the diagonal values are the square roots of the AVE for 11 variables, namely vegetation, water, diversity, rest, site, spatial perception, landscape preference, being away, extent, fascination, and mental restoration. The lower triangular matrix displays the Pearson correlation coefficients between variables. All absolute Pearson correlation coefficients between variables are less than the square root of the AVE, signifying robust discriminant validity among the latent variables (Bagozzi, 1981). The secondary approach is the heterotrait–monotrait criterion (HTMT), employed for a double check. The HTMT evaluates the genuine correlations among constructs by contrasting the mean of correlations both within and between constructs (Henseler et al., 2015). Table 9 shows that HTMT values were all below 0.85, meeting the suggested standard.

#### 3.2.5. Structural Model

This step was performed by assessing the PLS-SEM model for collinearity issues, assessing the significance of structural model relationships and assessing the predictive abilities of the model according to the coefficient of determination (R^2^ value), effect size *f*^2^ and Q^2^, and blindfolding (Table 10). Before hypothesis testing, it is necessary to diagnose the multicollinearity within the model. Fornell and Bookstein suggest assessing the degree of multicollinearity among variables in the model using the variance inflation factor (VIF) (Fornell & Bookstein, 1982). The multicollinearity risk was under control, since the variance inflation factors (VIFs) were within an accepted level of less than five.

We then assessed the significance of relationships among the variables, using the PLS algorithm and bootstrapping. Chin suggests that an R^2^ close to 0.670 indicates a high explanatory power, an R^2^ around 0.333 suggests moderate explanatory power, and an R^2^ around 0.190 indicates low explanatory power (Chin, 1998). With respect to the models’ predictive ability, the R^2^ of 0.243 and 0.370 can be considered a moderate level of predictive accuracy, and landscape preference explains 20.6% of the variance in perceived restorativeness at a low level. Chin and Cohen propose that the effect size of each path in structural equation models can be assessed based on *f*^2^ values; 0.020 < *f*^2^ < 0.150, 0.150 < *f*^2^ < 0.350, and *f*^2^ > 0.350 indicate a low, moderate, and high influence of exogenous variables on endogenous variables, respectively (Cohen, 2013). The results show that *f*^2^ (NC → LP) = 0.077 (low), *f*^2^ (AC → LP) = 0.035 (low), *f*^2^ (SA → LP) = 0.059 (low), *f*^2^ (LP → PR) = 0.259 (moderate), *f*^2^ (NC → MR) = 0.078 (low), *f*^2^ (AC → MR) = 0.003 (very low), *f*^2^ (SA → MR) = 0.010 (very low), *f*^2^ (LP → MR) = 0.010 (very low), and *f*^2^ (PR → MR) = 0.119 (low). For the assessment of predictive relevance in structural models, the non-parametric Stone–Geisser test can be employed, which utilizes a sample reuse technique called blindfolding to create residual estimates. Hair et al. (2011) argue that the predictive relevance Q^2^ of a structural model should be greater than 0, and the better the predictive relevance of the model being tested, the larger the Q^2^ (Hair et al., 2011). The results indicate that 0.082 ≤ Q^2^ (landscape preference, perceived restorativeness, mental restoration) ≤ 0.275, suggesting that the endogenous latent variables have good predictive relevance.

**Figure 9:**
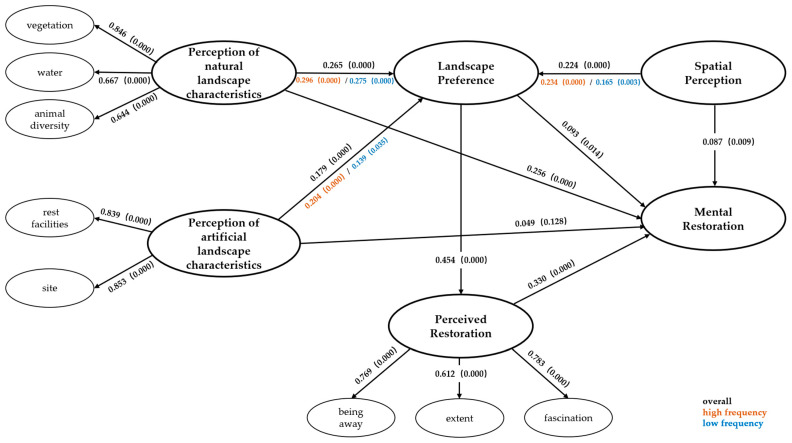
Results of the structural equation model.

With the help of bootstrapping algorithm in Smart PLS 4.0.8.5 software, the 95% bias correction and accelerated (BCa) bootstrap confidence intervals of 5000 bootstrap iterations were used for the test, and the results were obtained as shown in Table 11. To further explore the mediating effects of landscape preference and perceived restorativeness, we examined the indirect effects of the campus landscape perception on mental restoration with two mediators simultaneously (Figure 9). The findings reveal that both landscape preference and perceived restorativeness exert significant mediating effects (non-zero, as detailed in Table 12).

The results show that in the high-frequency group, the path coefficients of the perception of landscape characteristics on landscape preference are significantly higher than those in the low-frequency group. Specifically, in the NC → LP path, for the low-frequency group, β = 0.254, *p* < 0.001; for the high-frequency group, β = 0.298, *p* < 0.001. In the AC → LP path, for the low-frequency group, β = 0.119, *p* = 0.085; for the high-frequency group, β = 0.195, *p* < 0.001. In the SA → LP path, for the low-frequency group, β = 0.243, *p* < 0.001; for the high-frequency group, β = 0.204, *p* < 0.001. This indicates that long-term exposure strengthens the impact of perception on preference.

#### 3.2.6. Moderating Effect of the Objective Landscape Elements

Table 13 presents the results of the moderating effect test using the data obtained from image segmentation. It shows the effects of different landscape elements (architecture, sky, trees, etc.) on the relationships between landscape characteristic perception (natural, artificial, spatial) and landscape preference. Each row represents a specific interaction with the effect size and 95% confidence intervals provided, helping to clarify the moderating role of landscape elements.

The results revealed that the impact of NC on LP was significantly moderated by lawns (β = 0.089, 95% CI = [0.004, 0.172]), architecture (β = −0.106, 95% CI = [−0.207, −0.010]), and mulching (β = −0.100, 95% CI = [−0.176, −0.022]). Specifically, as the proportion of lawns increased, the positive influence of NC on LP was enhanced, while architecture and mulching had a dampening effect on this relationship.

Similarly, SA exhibited a variable impact on LP, which was influenced by the presence of specific landscape elements. For instance, trees (β = −0.071, 95% CI = [0.005, 0.129]), mulching (β = 0.116, 95% CI = [0.042, 0.179]), and roads (β = −0.091, 95% CI = [−0.164, −0.013]) were found to moderate the relationship between SA and LP. This suggests that the effect of SA on LP is not static but contingent upon the configuration of these landscape elements.

## 4. Discussion

### 4.1. Effects of Landscape Characteristics on Landscape Preference

Our results suggest that the perceived natural, artificial, and spatial configuration of CGS landscape characteristics all significantly influence landscape preference. This finding not only corroborates the views of previous scholars (Q. Liu et al., 2018; K. Li, 2024) but also extends our understanding of how different landscape dimensions collectively shape students’ landscape preference. By integrating natural, artificial, and spatial elements into a single framework, this study provides a more comprehensive perspective on the complexity of landscape preference formation in campus environments.

Notably, natural landscape characteristics exert the strongest influence on landscape preference, implying that landscapes resembling natural settings are more likely to be preferred and are deemed restorative (Hagerhall et al., 2004). The findings revealed a nuanced interaction between the perception of natural landscape characteristics and specific landscape elements in influencing landscape preference. The positive impact of natural characteristics on preference was found to be enhanced by the presence of lawns, suggesting that CGSs can amplify the attractiveness of natural elements. Conversely, architecture and mulching were found to have a dampening effect, indicating that excessive built structures or ground coverings may diminish the restorative potential of natural landscapes. This implies that campus landscape design should carefully balance natural and artificial elements to maximize their combined benefits for students’ mental health.

The impact of artificial landscape characteristics on preference levels, though less researched, is an important aspect that deserves attention. In our study, artificial elements such as open space amenities were found to contribute to students’ landscape preference. College students who frequently visit campus green spaces exhibit a stronger perception–preference correlation, and this finding indicates that long-term exposure to campus greenery can enhance the influence of perception on preference. The comfort of seating and its orientation can significantly influence students’ willingness to stay and engage with the environment, thereby affecting their landscape preference. This suggests that the design of artificial elements should not only focus on esthetics but also on functionality and user experience to enhance their positive impact on students’well-being.

A clean and sanitary environment can be considered as a marker of perceived safety. In public spaces, people’s spatial preference cognition is associated with perceptions of fear/safety. Clean environments reduce the risk of disease transmission and attenuate perceived health threats, thereby enhancing users’ comfort and sense of security (Dessauvagie et al., 2022). This finding aligns with existing research demonstrating that environmental cleanliness and hygiene conditions influence user preferences. Future campus landscape design should focus on the multi-functional nature of artificial landscape features. Specifically, incorporating clean and sanitary facilities, providing comfortable seating, and implementing ergonomic path design can significantly enhance college students’ preferences and length of stay.

Moreover, the cultivation of preference may entail a deeper emotional experience or engagement. In terms of the spatial perception dimension of the campus environment, the spatial perception of green space is closely related to human emotions. Emotion usually serves as the internal driving force for landscape preference, suggesting that people’s preference for the environment stems from a resonance with spatial perception—a kind of deep-level emotional communication (Q. Liu et al., 2017). This could explain why we found that the perception of artificial elements is less preferred than spatial perception, and the underlying reasons warrant further investigation.

### 4.2. Effects of Landscape Preference on Perceived Restorativeness

Our study also reveals that landscape preference significantly impacts environmental perceived restorativenesss. This indicates that individuals who have a stronger preference for an environment also report a higher level of perceived restorativenesss from it, aligning with the nativity hypothesis. Previous research has shown that the greater people’s preference for green space environments, the more restorative they are perceived to be. Thus, the effect of students’ preferences for campus landscapes on perceived restorativeness is consistent with the majority of current research (K. M. Korpela et al., 2001; Q. Liu et al., 2017).

### 4.3. Effects of Landscape Characteristics on Mental Restoration

The direct effect of landscape characteristic perception on mental restoration is also consistent with the results of several studies. Notably, natural landscape characteristics enhance mental restoration (*p* = 0.000 < 0.001), corroborating the findings of previous studies (Q. Liu et al., 2018; Zhou et al., 2024). The presence of diverse plant and animal species, clear water bodies, and other natural elements in the environment is more conducive to engaging with nature for mental restoration (Whitburn et al., 2020). Additionally, the effect of spatial feature perception on mental restoration is also significant (*p* = 0.000 < 0.001). The spatial perception represents a deeper level of people’s psychological and behavioral emotional expression of a certain environment, essentially a sense of place identity (Li et al., 2023). This deep emotional connection is key to recovery, akin to Kaplans’ attentional recovery theory. It is worth mentioning that among these environmental features, the perception of artificial landscape characteristics does not significantly impact psychological recovery. This may be due to the fact that features like winding paths and undulating terrain are merely visually perceived without evoking a strong emotional response. These common basic artificial elements, abundant in the environment, do not directly impact psychological recovery, as they fail to appeal to students. It suggests that functional aspects like artificial landscape characteristics only meet external needs, and true mental restoration requires a deeper emotional connection. Therefore, it can be inferred that artificial feature perception does not affect mental restoration, implying environmental feature perception does not directly lead to mental restoration.

### 4.4. Explanation of Mediation Effects

The mediation analysis reveals that the perception of campus landscape characteristics in promoting mental restoration is mediated by landscape preference and perceived restorativeness. From an environmental psychology perspective, this finding implies that perceiving restorative campus environments is a sequential psychological process, involving a cognitive evaluation of the environment, emotional response, and subsequent emotional activation. A more naturalized green space environment is more likely to evoke landscape preference among students. Preferred environments offer an escape from the hustle and bustle of daily life, aligning with perceived restorativeness and supporting the restoration of health and well-being. For instance, animals are generally favored for their esthetic and ecological values. The perception of a rich diversity of animals can attract and invigorate individuals. This visual and auditory proximity to nature can alleviate the monotony of life, exerting a positive influence (Marselle et al., 2016). Therefore, it can be proposed that restorative environments typically feature natural elements such as vegetation, water, animals, and serenity (Van den Berg, 2005). However, the restorative effects are a result of the interplay between the surrounding environment, individual mental states, and the interaction between people and their surroundings.

### 4.5. Explanation of Moderating Effects

Campus landscape elements exhibit a positive moderating effect on localized pathways within the relationship between landscape perception and preference. This result aligns with ART, which emphasizes the restorative capacity of natural environments (R. Kaplan et al., 1989). However, the moderating effects vary across psychological pathways. For example, mulching demonstrates a negative moderating effect. According to Falk and Balling (2009), humans’ innate preference for savanna-like landscapes may stem from evolutionary adaptations, as open settings allowed for timely responses to threats concealed by vegetation. This suggests that emotional responses to natural features may be linked to subconscious safety and survival mechanisms.

The correlation analysis across four spatial types (waterfront, vegetation, squares, and courtyards) revealed nuanced moderating effects of landscape element arrangements on the perception–preference relationship. Specifically, in waterfront spaces, the presence of water bodies significantly amplified the positive correlation between perceived natural landscapes and preferences, while architectural elements weakened perceived naturalness and serenity. This suggests that the spatial configuration of elements—rather than their mere presence—plays a critical moderating role. In other words, the moderating effect of landscape elements is contingent on spatial typology. For example, while trees universally enhanced natural feature perception, their moderating effect was strongest in vegetation spaces where architectural interference was minimal. These findings advocate for context-sensitive design, in which optimizing element configurations (e.g., minimizing roads in squares, maximizing water visibility in courtyards) can amplify restorative outcomes.

### 4.6. Limitations

Summarizing the study, the results establish the intricate link between campus landscape characteristics, landscape preference, perceived restorativeness, and mental health, providing a landscape design perspective on the pathway’s formation and its implications for design practices. Additionally, the study confirms the significance of campus environments for students’ psychological well-being and enhances our comprehension of how environmental perception influences health. Nevertheless, the study has limitations: while the research conducted in campus natural settings faced uncontrollable variables like temperature and noise despite controlling for site factors, the campus landscape evaluation scale, also developed due to a paucity of existing scales, may not capture all site characteristics, potentially lacking comprehensive evaluation dimensions. This could lead to an over-simplification or bias in indicator selection. While campus perceived restorativeness involves a complex pathway, our analysis was preliminary. A deeper dive into the factors influencing this pathway, including influencing elements and mechanisms of perceived restorativeness, is advised for future studies. Finally, similar to most studies, our research focuses on the immediate restorative effects, yet there is relatively little investigation into the cumulative effects on long-term health, such as the enhancement of well-being due to an increase in the visiting frequency. In terms of exploring the mechanism, it is recommended to employ longitudinal studies and intervention experimental designs to thoroughly explore the causal relationship between the campus environment and the mental health of college students.

## 5. Conclusions

This study employed a comprehensive structural equation model to unravel the complex interplay between campus landscape characteristics, psychological factors, and mental restoration among university students. By incorporating multiple dimensions of landscape perception and their underlying psychological mechanisms, we aimed to provide a holistic understanding of how campus environments can be optimized to support students’ psychological well-being. The integration of advanced deep learning techniques allowed for an efficient and accurate extraction of landscape characteristics from a vast collection of on-site images. This innovative methodological approach not only enhanced the objectivity of data collection but also provided a scalable solution for future landscape research and design applications. The integration of perceptive qualities and landscape properties facilitated a more in-depth understanding of environmental restoration, backed by reliable estimations. Regarding the seven hypotheses, H1 can be supported. The results indicated that natural landscape characteristics, artificial landscape characteristics, and spatial perception presented a significant contribution to landscape preference. H2 can be supported in that landscape preference presented a significant contribution to perceived restorativeness. H3 can be supported in that perceived restorativeness presented a significant contribution to mental restoration. H4 can be supported in that landscape preference presented a significant contribution to mental restoration. H5 can be partially supported. The result affirmed that natural landscape characteristics and spatial perception presented a significant contribution to mental restoration, whereas the effect of artificial landscape characteristics on mental restoration was insignificant. H6 can be supported in that the mediating effect of preference and perceived restorativeness between landscape characteristic perception and mental restoration was significant. H7 can be partially supported: specific landscape elements (e.g., lawns) positively moderated the relationship between landscape perception and preference, whereas buildings and vegetation cover weakened the influence of natural landscape perception on preference. Furthermore, the moderating effect of landscape elements is contingent on spatial typology. These findings shed new light on the complex process in environmental restoration, in which psychological and physical factors are intertwined.

## Figures and Tables

**Table 1:** General description of the study site.

School	Location	Date of Establishment	Size and Population	Type of University District	Number of Sample Sites	Space Type
Nanjing University of Science and Technology	Xuanwu district	1953	2,133,344 m^2^ 28,000 people	O	7	WS, VS, SS, CS
Nanjing University of Finance and Economics (Qixia Campus)	Qixia district	2003	1,000,005 m^2^ 20,000 people	N	8	WS, VS, SS, CS
Nanjing Forestry University	Xuanwu district	1955	837,997.19 m^2^ 25,000 people	O	8	WS, VS, CS
China Pharmaceutical University (Jiangning Campus)	Jiangning district	2012	1,333,340 m^2^ 13,000 people	N	7	WS, VS, SS, CS
Nanjing Audit University	Pukou district	2003	1,080,005.4 m^2^ 15,000 people	N	2	SS, WS
Nanjing University of Engineering	Jiangning district	2005	1,700,008.5 m^2^ 24,000 people	N	4	WS, VS, SS, CS

Note: N, new; O, old; WS, waterfront space; VS, vegetation space; SS, square; CS, courtyard.

**Table 2:** Summary of outcome construct and perceptual factors.

Construct		Item	Reference
Landscape Preference		LP1: This place has high quality of naturalness.	Mangone et al. (2021); Ode et al. (2009)
		LP2: The landscape is of quality of diversity	
		LP3: This place has high quality of planning and design.	
		LP4: The landscape is of liveliness and good visual order.	
		LP5: This is a fascinating place.	
		LP6: In overall, this place has quality of aesthetic appeal.	
Mental Restoration		MR1: How would you rate the improvement in self-perceived energy levels after this CGS visit?	Zhou et al. (2022); H. Liu et al. (2017)
		MR2: How would you rate the improvement in self-perceived health status after this CGS visit?	
		MR3: How would you rate the improvement in self-perceived confidence after this CGS visit?	
		MR4: To what extent do you feel that this CGS visit relaxed you?	
		MR5: To what extent do you feel that this CGS visit restored your mood?	
Perceived restorativeness	PR1: Being away	PR1-1: There’s a different vibe here.	Hartig et al. (1997)
		PR1-2: I feel really detached from my daily routine.	
		PR1-3: Being here is an escape experience.	
		PR1-4: I can relax here.	
		PR1-5: I feel really detached from the stress of everyday life.	
	PR2: Extent	PR2-1: I’m confused here (reversed item).	
		PR2-2: There is too much going on (reversed item).	
		PR2-3: There is a great deal of distraction here (reversed item).	
	PR3: Fascination	PR3-1: Places like that are fascinating.	
		PR3-2: My attention is drawn to many interesting things.	
		PR3-3: I feel really drawn to details in this place.	
		PR3-4: I would like to spend more time looking at the surroundings.	

**Table 3:** Results of exploratory factor analysis and examination.

Factors	Factor Loadings						
	Factor 1	Factor 2	Factor 3	Factor 4	Factor 5	Factor 6	Communality
NC1-1 Large area of lawn coverage	0.848						0.738
NC1-2 Rich plant species	0.871						0.810
NC1-3 Vertical layers of plants are rich	0.842						0.714
NC1-4 Natural planting form	0.813						0.705
NC1-5 Good plant shade	0.837						0.714
NC2-1 Large waterfront					0.785		0.696
NC2-2 Good water quality					0.787		0.728
NC2-3 Water landscape with good hydrophilicity					0.804		0.697
NC3-1 Many animals						0.868	0.815
NC3-2 Natural sounds such as cicadas and birds can be heard						0.859	0.806
AC1-1 Adequate rest facilities				0.820			0.748
AC1-2 Rest facilities are more comfortable				0.848			0.802
AC1-3 Rest facilities are oriented towards the scenery				0.752			0.711
AC2-1 Winding roads			0.888				0.840
AC2-2 Varied terrain			0.727				0.664
AC2-3 The site is clean and hygienic			0.821				0.753
SA1 Feels natural (naturalness)		0.722					0.603
SA2 Feels calm (serenity)		0.841					0.735
SA3 Feels safe (sheltered)		0.777					0.618
SA4 The site is suitable for students to interact socially (social)		0.859					0.755
Eigenvalue	4.642	3.390	2.220	1.612	1.559	1.227	
Variance explained (%)	19.291	13.334	11.466	10.891	10.080	8.191	
Cumulative variance explained (%)	19.291	32.625	44.091	54.982	65.062	73.253	

Note: Only factor loadings above 0.50 are listed.

**Table 4:** Reliability analysis of the questionnaire.

Variables	Item	CITC	Cronbach’s α Values After Deletion of Terms	Cronbach’s α Values for Each Variable
Vegetation	NC1-1	0.788	0.881	0.906
	NC1-2	0.815	0.875	
	NC1-3	0.749	0.889	
	NC1-4	0.733	0.892	
	NC1-5	0.743	0.890	
Water	NC2-1	0.605	0.607	0.740
	NC2-2	0.569	0.651	
	NC2-3	0.524	0.706	
Animal Diversity	NC3-1	0.553	—	0.712
	NC3-2	0.553	—	
Rest	AC1-1	0.590	0.782	0.800
	AC1-2	0.732	0.632	
	AC1-3	0.618	0.757	
Site	AC2-1	0.743	0.661	0.813
	AC2-2	0.570	0.838	
	AC2-3	0.684	0.721	
Spatial Perception	SA1	0.589	0.810	0.826
	SA2	0.721	0.749	
	SA3	0.595	0.806	
	SA4	0.711	0.753	

**Table 5:** Summary statistics (N = 759).

Measures	Measure Types	N	%
Gender	Male	386	50.9
	Female	373	49.1
Education	Undergraduate	401	52.8
	Master student	286	37.7
	Doctoral student	72	9.5
Have a design background	Yes	327	43.1
	No	432	56.9
Hours spent on studying (classes, revision, and homework) in the last week	Average of 1–2 h per day	173	22.8
	Average of 3–4 h per day	237	31.2
	Average of 5–6 h per day	295	38.9
	Average of 7 h and more per day	54	7.1
Hours of sleep per day for the last week	Average of 10 h and more per day	32	4.2
	Average of 8–9 h per day	344	45.3
	Average of 6–7 h per day	333	43.9
	Average of 4–5 h per day	50	6.6
Self-stress evaluation	No pressure	52	6.9
	Not much stress	224	29.5
	Occasional bad moods	281	37
	Often feel negative and depressed	162	21.3
	Stress is very high and seriously affects life	40	5.3
Whether there will be problems with concentration	Hardly ever	16	2.1
	Less frequently	148	19.5
	Occasionally	303	39.9
	Often	243	32.0
	Always	49	6.5
When you have problems with stress, inattention, etc., do you want to make changes?	No, I don’t want to make a change	47	6.2
	Maybe, but I don’t know by what means	419	55.2
	Yes, I can find a way to self-regulate	293	38.6
Frequency of activities on CGSs	3–5 times a week and above	58	7.6
	2–3 times a week	187	24.6
	About 5 times a month	363	47.8
	Almost not	151	19.9
Time spent in CGSs	15 min	318	41.9
	16–30 min	324	42.7
	31 min–1 h	89	11.7
	More than 1 h	28	3.7
How many people usually go to CGSs together	Alone	168	22.1
	2–3 persons	503	66.3
	4–6 persons	62	8.2
	7 and above	26	3.4

**Table 6:** Descriptive statistics of the variables (N = 759).

Second-Order Variable	First-Order Variable	Mean	Std.	Minimum	Maximum
Perception of natural landscape characteristics	Vegetation	3.43	1.77	1.00	7.00
	Water	3.90	1.50	1.00	7.00
	Animal diversity	3.71	1.57	1.00	7.00
Perception of artificial landscape characteristics	Rest	3.64	1.54	1.00	7.00
	Site	3.70	1.48	1.00	7.00
Spatial perception		4.78	1.30	1.00	7.00
Landscape preference		4.73	1.28	1.00	7.00
Mental restoration		4.66	1.32	1.50	7.00
Perceived restorativeness	Being away	4.42	1.44	1.20	7.00
	Extent	4.80	1.26	1.00	7.00
	Fascination	4.22	1.42	1.25	7.00
Landscape elements	Architecture	11.31	12.74	43.90	0.00
	Sky	18.56	13.40	43.55	0.00
	Trees	22.80	12.17	62.34	4.60
	Lawns	9.96	12.17	39.33	0.00
	Mulching	10.13	8.12	25.59	0.00
	Water	5.91	9.44	28.32	0.00
	Roads	6.83	9.50	41.70	0.00

Note: Normal fonts are latent variables, and italics are the observed variables.

**Table 7:** Reliability and convergent validity test of the initial model.

First-Order	Item	Loading	Cronbach’s α	C.R.	AVE	Second-Order	Loading	Cronbach’s α	C.R.	AVE
NC1	NC1-1	0.853	0.903	0.928	0.722	NC	0.846	0.810	0.766	0.525
	NC1-2	0.887								
	NC1-3	0.806								
	NC1-4	0.852								
	NC1-5	0.847								
NC2	NC2-1	0.865	0.803	0.881	0.713		0.667			
	NC2-2	0.888								
	NC2-3	0.775								
NC3	NC3-1	0.836	0.722	0.874	0.777		0.644			
	NC3-2	0.925								
AC1	AC1-1	0.815	0.801	0.883	0.715	AC	0.839	0.816	0.834	0.716
	AC1-2	0.876								
	AC1-3	0.845								
AC2	AC2-1	0.905	0.826	0.896	0.743		0.853			
	AC2-2	0.832								
	AC2-3	0.847								
SA	SA1	0.834	0.829	0.884	0.657					
	SA2	0.886								
	SA3	0.714								
	SA4	0.800								
LP	LP1	0.891	0.921	0.940	0.760					
	LP2	0.834								
	LP3	0.876								
	LP4	0.888								
	LP6	0.867								
MR	MR1	0.904	0.917	0.938	0.753					
	MR2	0.853								
	MR3	0.818								
	MR4	0.889								
	MR5	0.871								
PR1	PR1-1	0.858	0.920	0.940	0.759	PR	0.769	0.872	0.767	0.526
	PR1-2	0.902								
	PR1-3	0.875								
	PR1-4	0.839								
	PR1-5	0.880								
PR2	PR2-1	0.874	0.813	0.889	0.727		0.612			
	PR2-2	0.850								
	PR2-3	0.832								
PR3	PR3-1	0.880	0.923	0.945	0.812		0.783			
	PR3-2	0.899								
	PR3-3	0.918								
	PR3-4	0.906								

Note: NC1 to NC3 refer to the sub-construct of NC; AC1 and AC2 refer to the sub-construct of AC; PR1 to PR3 refer to the sub-construct of PR.

**Table 8:** Discriminant validity of constructs using the Fornell–Larcker criterion.

	AC1	AC2	NC1	NC2	NC3	PR1	PR2	PR3	MR	LP	SA
AC1	0.846										
AC2	0.432	0.862									
NC1	0.158	0.297	0.849								
NC2	0.270	0.271	0.276	0.844							
NC3	0.137	0.231	0.374	0.276	0.882						
PR1	0.28	0.143	0.132	0.154	0.187	0.871					
PR2	0.189	0.171	0.142	0.198	0.144	0.257	0.852				
PR3	0.253	0.255	0.297	0.361	0.252	0.303	0.342	0.901			
MR	0.263	0.27	0.344	0.395	0.277	0.349	0.288	0.476	0.867		
LP	0.298	0.275	0.264	0.358	0.292	0.241	0.418	0.385	0.391	0.871	
SA	0.262	0.21	0.221	0.225	0.194	0.251	0.222	0.354	0.333	0.349	0.811

Note: PR, perceived restorativeness; LP, landscape preference; NC, perception of natural landscape characteristics; AC, perception of artificial landscape characteristics; SA, spatial perception; MR, mental restoration.

**Table 9:** Discriminant validity of constructs using the HTMT criterion.

	AC1	AC2	NC1	NC2	NC3	PR1	PR2	PR3	MR	LP	SA
AC1											
AC2	0.527										
NC1	0.182	0.342									
NC2	0.344	0.324	0.295								
NC3	0.171	0.299	0.445	0.316							
PR1	0.324	0.164	0.145	0.188	0.232						
PR2	0.23	0.202	0.161	0.234	0.183	0.29					
PR3	0.29	0.291	0.325	0.409	0.303	0.328	0.39				
MR	0.302	0.309	0.379	0.457	0.337	0.378	0.323	0.517			
LP	0.341	0.31	0.288	0.41	0.358	0.262	0.476	0.412	0.422		
SA	0.301	0.245	0.237	0.237	0.228	0.283	0.252	0.384	0.366	0.382	

**Table 10:** Coefficient of determination–predictive relevance–utility value–variance inflation factor.

Indicators	R^2^ Value	Adj. R^2^	Q^2^ Value	*f*^2^ Value					VIF				
Constructs	—	—	—	NC	AC	SA	LP	PR	NC	AC	SA	LP	PR
MR	0.370	0.366	0.275	0.078	0.003	0.010	0.010	0.119	1.336	1.276	1.257	1.428	1.456
PR	0.206	0.205	0.082	—	—	—	0.259	—	—	—	—	1.000	—
LP	0.243	0.240	0.180	0.077	0.035	0.059	—	—	1.206	1.202	1.130	—	—

**Table 11:** Summary of the detailed direct effects.

Hypothesis	Path	Effect	Std.	t	*p*	95% CI	Bias-Corrected 95% CI	Test Result
H1a	NC → LP	0.265	0.034	7.823	<0.001 ***	[0.197, 0.333]	[0.198, 0.334]	Supported
H1b	AC → LP	0.179	0.037	4.819	<0.001 ***	[0.104, 0.249]	[0.103, 0.249]	Supported
H1c	SA → LP	0.224	0.034	6.516	<0.001 ***	[0.158, 0.292]	[0.155, 0.290]	Supported
H2	LP → PR	0.454	0.032	14.151	<0.001 ***	[0.391, 0.515]	[0.388, 0.512]	Supported
H3	PR → MR	0.330	0.037	8.964	<0.001 ***	[0.259, 0.403]	[0.255, 0.400]	Supported
H4	LP → MR	0.093	0.038	2.470	0.014 **	[0.020, 0.168]	[0.022, 0.171]	Supported
H5a	NC → MR	0.256	0.034	7.496	<0.001 ***	[0.187, 0.325]	[0.187, 0.324]	Supported
H5b	AC → MR	0.049	0.032	1.524	0.128 ^ns^	[−0.014, 0.113]	[−0.015, 0.111]	Not supported
H5c	SA → MR	0.087	0.034	2.603	0.009 ***	[0.021, 0.153]	[0.025, 0.156]	Supported

Note: ns = *p* > 0.05; ** = *p* < 0.05; *** = *p* < 0.01.

**Table 12:** Summary of the full model with mediating effects.

Hypothesis	Path	Effect	95% CI	Bias-Corrected 95% CI	Test Result
H6a	NC → LP → PR → MR	0.040	[0.026, 0.057]	[0.026, 0.057]	Supported
H6b	AC → LP → PR → MR	0.027	[0.015, 0.040]	[0.015, 0.041]	Supported
H6c	SA → LP → PR → MR	0.034	[0.021, 0.049]	[0.020, 0.049]	Supported

**Table 13:** Landscape elements’ moderating effects on perception of landscape characteristics—landscape preference relationships.

Hypothesis	Path	Effect	95% CI
H7	Lawn * NC → LP	0.089	[0.004, 0.172]
	Sky * NC → LP	0.016	[−0.078, 0.122]
	Trees * NC → LP	0.055	[−0.024, 0.133]
	Architecture * NC → LP	−0.106	[−0.207, −0.010]
	Mulching * NC → LP	−0.100	[−0.176, −0.022]
	Water * NC → LP	0.030	[−0.056, 0.117]
	Road * NC → LP	0.033	[−0.048, 0.109]
	Lawn * AC → LP	0.063	[−0.032, 0.154]
	Sky * AC → LP	−0.058	[−0.157, 0.033]
	Trees * AC → LP	−0.063	[−0.139, 0.009]
	Architecture * AC → LP	−0.024	[−0.113, 0.064]
	Mulching * AC → LP	0.028	[−0.062, 0.108]
	Water * AC → LP	−0.028	[−0.117, 0.057]
	Road * AC → LP	−0.007	[−0.093, 0.078]
	Lawn * SA → LP	0.081	[−0.009, 0.179]
	Sky * SA → LP	−0.039	[−0.127, 0.054]
	Trees * SA → LP	0.071	[0.005, 0.129]
	Architecture * SA → LP	−0.015	[−0.102, 0.079]
	Mulching * SA → LP	0.116	[0.042, 0.179]
	Water * SA → LP	0.050	[−0.022, 0.129]
	Road * SA → LP	−0.091	[−0.164, −0.013]

Note: The items with * represent interaction terms.

## Data Availability

The raw data in this article will be available upon request.

## Appendix A

**Table A1:** Percentage of visual landscape elements in the 36 study sample sites.

Image Name	Architecture (%)	Sky (%)	Trees (%)	Lawn (%)	Mulching (%)	Water (%)	Roads (%)
1	3.66	30.37	13.36	22.58	25.59	0.00	3.78
2	0.77	25.47	22.01	0.12	0.00	22.06	0.00
3	9.11	31.52	4.66	0.00	21.67	15.85	14.59
4	32.74	10.16	12.81	0.00	17.58	0.00	0.00
5	0.02	32.26	25.42	34.40	2.12	0.00	0.22
6	2.54	22.63	20.40	0.00	6.76	26.97	20.14
7	19.15	21.78	4.60	0.00	9.79	0.00	41.70
8	43.90	2.12	8.20	27.63	14.54	0.00	0.00
9	0.00	2.10	52.14	8.40	20.22	0.00	13.80
10	2.41	36.33	18.19	23.17	10.32	3.14	6.16
11	1.64	34.06	18.70	0.00	2.10	0.00	0.00
12	29.48	12.98	16.80	2.54	12.15	0.00	17.90
13	12.84	23.88	14.02	26.92	1.03	0.00	0.04
14	5.41	29.52	25.12	0.00	22.17	16.49	0.00
15	5.06	37.92	8.14	1.63	0.00	0.00	0.00
16	21.80	8.15	5.19	28.88	12.24	6.20	15.50
17	1.17	21.74	32.83	19.67	21.90	0.00	0.00
18	0.79	0.08	51.21	0.04	0.00	18.32	4.11
19	0.08	22.82	27.56	6.37	5.59	0.00	0.13
20	35.52	2.10	26.88	0.00	20.81	0.00	12.10
21	27.65	0.00	32.38	39.33	0.02	0.00	0.41
22	35.02	0.02	18.62	3.57	0.00	15.51	11.86
23	21.32	18.35	18.65	1.09	0.01	0.00	0.00
24	2.45	12.33	36.19	4.17	7.32	0.00	26.16
25	9.80	4.92	28.48	0.04	17.10	0.00	0.03
26	0.00	22.80	28.09	0.00	25.55	21.82	0.00
27	2.24	23.46	22.32	0.55	3.90	0.00	0.00
28	18.64	25.06	18.70	9.50	8.62	0.00	11.00
29	0.10	3.34	62.34	19.95	3.67	0.00	9.82
30	0.00	43.55	19.97	1.42	0.00	16.82	0.00
31	6.79	0.08	26.21	18.04	10.00	28.32	6.11
32	30.79	0.00	15.21	0.04	12.80	0.00	24.11
33	1.23	34.03	16.49	31.40	16.01	0.00	0.00
34	3.79	20.08	17.21	8.04	15.72	23.32	4.80
35	3.83	41.54	22.85	5.15	11.23	0.00	0.00
36	9.55	13.23	42.08	21.69	6.15	0.00	6.95
